# Anterior Reduction, Discectomy, and Three Cortical Iliac Bone Grafting With Instrumentation to Treat A Huge Tear Drop Fracture of the Axis: A Case Report and Literature Review

**DOI:** 10.1097/MD.0000000000003376

**Published:** 2016-04-18

**Authors:** Litai Ma, Yi Yang, Quan Gong, Chen Ding, Hao Liu, Ying Hong

**Affiliations:** From the Department of Orthopaedics (LM, YY, QG, CD, HL), and Operation Room, West China Hospital, Sichuan University, Chengdu, Sichuan Province, China (YH).

## Abstract

Fractures of the axis body have been little reported and treatment strategies remain controversial and individualized. Not more than 10 cases of huge tear drop fracture of the axis (HTDFA) have been reported in previous studies and the treatment method varies from conservative treatment to an anterior, or posterior, approach surgery. Considering the sparse knowledge of HTDFA, we present a special case report to share our experience and to explore the safety and effectiveness of anterior reduction and fusion to treat HTDFA.

A 24-year-old man was referred to our department; he presented with neck pain lasting for 12 h since being involved in a roll-over motor vehicle accident. His neck movement was limited but there was no neurological compromise. Physical examination of the patient showed myodynamia of four limbs Grade 5, Hoffmann sign (–), and Babinski sign (–). Three-dimensional reconstruction computed tomography (CT) confirmed a huge tear drop fracture of the anterior–inferior corner of the axis and discontinuity of the cortex of the axis. After discussion with the spinal surgeon team in the department and an effective conversation with the patient, surgery involving anterior reduction, discectomy, and three cortical iliac bone grafts with instrumentation after transnasal induction of general anesthesia was performed. The patient was instructed to wear a cervical collar until he returned to our department for a follow-up examination some 3 months after surgery. The 3-month postoperative x-ray and CT scan showed a good position of the implant and bony fusion at the C2/3 segment.

Anterior reduction, discectomy, and three cortical iliac bone grafts with instrumentation to treat HTDFA are effective, safe, and simple. Of course, longer follow-up duration and more cases are warranted to verify this procedure. Anterior reduction, discectomy, and bone grafting with instrumentation are warranted for most HTDFA cases. However, if HTDFA incorporates other complex fractures, such as fracture of the posterior structure, an anterior and posterior union surgery is recommended.

## INTRODUCTION

Cervical spine injuries are reported to occur in 3% to 4% of all trauma patients and among them there are ∼12,000 people associated with spinal cord injury *per annum* in the United States.^1^ According to previous reports, fractures of the axis account for 17% to 27% of all cervical spine injuries.^2,3^ Fractures of the axis can be divided into three clinically relevant categories: odontoid fractures, hangman's fracture injuries (traumatic spondylolisthesis), and fractures of the axis body, involving all other fracture injuries to the axis vertebra.^4^ Fractures of the odontoid process and hangman fractures are the most common types of axis fractures and are reported in a series of recent studies. However, fractures of the axis body have been little reported and the treatment strategies remain controversial and individualized. Tear drop fracture, characterized by the separation and downward and forward displacement of the anterior inferior margin of the involved vertebral body, was first described by Kahn and Schneider in 1956.^5^ Tear drop fracture of the axis is rarely seen and it accounts for only 1% to 3% of all cervical spine fractures.^6^ The exact definition of a huge tear drop fracture of the axis (HTDFA) remains controversial; however, if the fracture line lies over half of lower endplate of the axis in the sagittal plane, it is often regarded as “huge” as described by Yang et al in their case report.^7^ A small tear fracture of the axis is often treated by nonoperative method such as immobilization by plaster casts or orthoses, or skull traction. However, the treatment method adopted for HTDFA remains so controversial that a lot of spinal surgeons are confused. Not more than 10 cases of HTDFA were reported in previous studies and the treatment method varies from conservative treatment to anterior or posterior approach surgery. Considering the paucity of knowledge of HTDFA, we present this special case report to share our experience and to explore the safety and effectiveness of anterior reduction and fusion to treat HTDFA with a minor follow-up some 3 months later. More importantly, previous cases were reviewed and the surgical strategies adopted were discussed.

### Case Description

The patient provided informed consent for the publication of their clinical and radiological data. This case report was approved by the Medical Ethical Committee of West China Hospital, Sichuan University.

**FIGURE 1:**
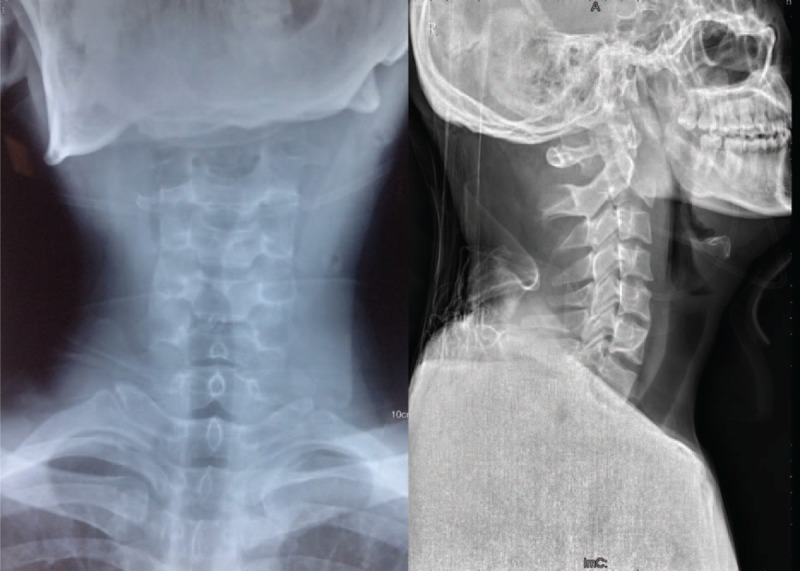
Preoperative anteroposterior and lateral x-rays revealed huge tear drop fracture of anterior–inferior corner of the axis.

**FIGURE 2:**
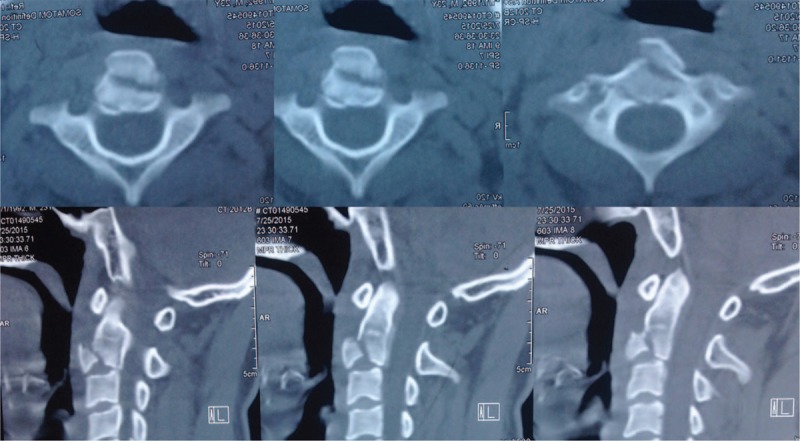
Preoperative three-dimensional reconstruction CT confirmed huge tear drop fracture of anterior-inferior corner of the axis, and the discontinuity of the cortex thereof. CT = computed tomography.

**FIGURE 3:**
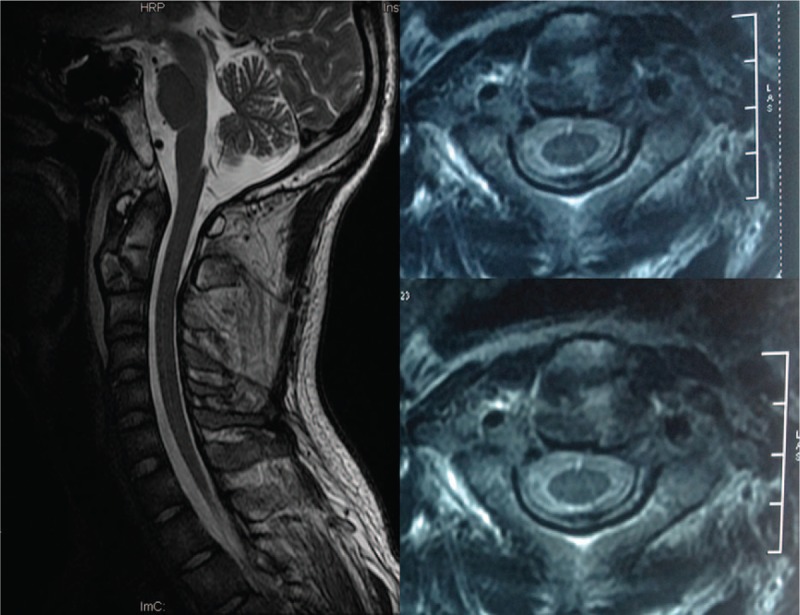
Preoperative cervical MRI showed hyperintensity in C2/3 intervertebral disc and pre-cervical soft tissue. MRI = magnetic resonance imaging.

A 24-year-old man was referred to our department who presented with neck pain that had lasted for 12 h since his involvement in a roll-over motor vehicle accident. His neck movement was limited but there was no neurological compromise. The physical examination of the patient showed myodynamia of four limbs Grade 5, Hoffmann sign (–), and Babinski sign (–). The sensory function of trunk and limbs was normal and symmetrical. Cervical x-rays revealed a huge tear drop fracture of the anterior–inferior corner of the axis (Figure 1). Three-dimensional reconstruction computed tomography (CT) confirmed the huge tear drop fracture of the anterior–inferior corner of the axis, and the discontinuity of the cortex of the axis (Figure 2). Cervical magnetic resonance imaging (MRI) showed hyperintensity in the C2/3 intervertebral disc and pre-cervical soft tissue (Figure 3).

**FIGURE 4:**
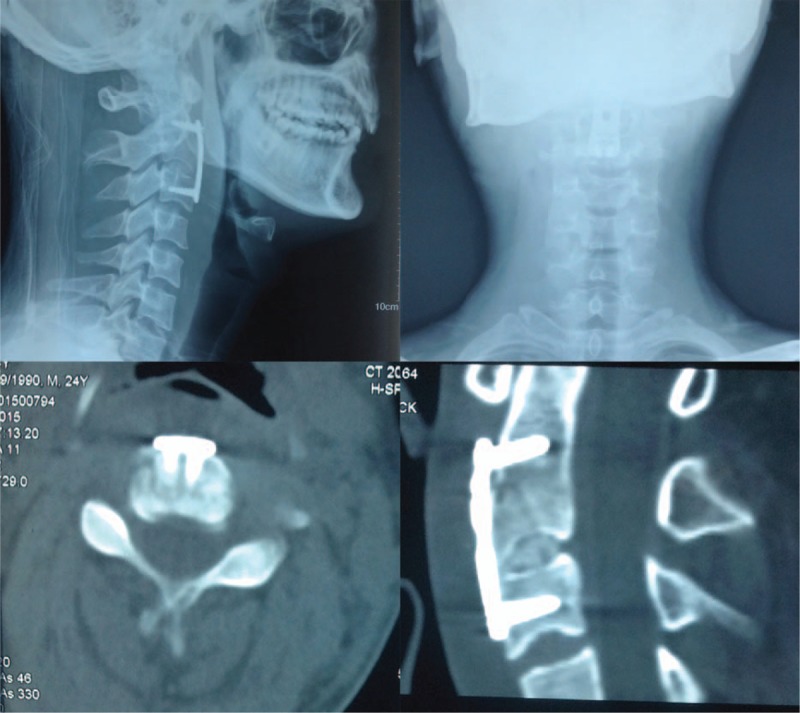
One day postoperative x-ray and CT scan showed a good position of the huge tear drop fracture fragment, three cortical iliac bones, and the anterior cervical plate. CT = computed tomography.

**FIGURE 5:**
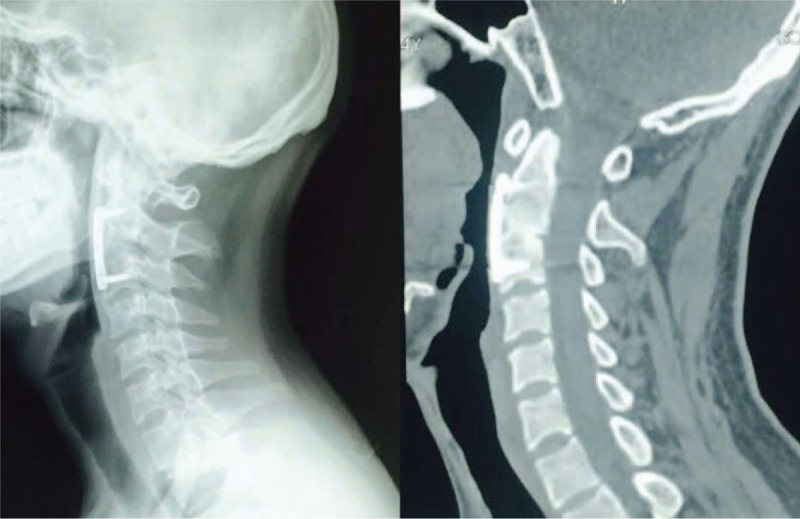
Three months postoperative x-ray and CT scan showed a good position of the implant and bony fusion at the C2/3 segment. CT = computed tomography.

After discussion with the spinal surgery team in the department and an effective conversation with the patient, a surgical plan involving anterior reduction, discectomy, and three cortical iliac bone grafts with instrumentation was planned. The surgery was performed using a classical anterior approach by an experienced surgeon after transnasal induction of general anesthesia on 31 July 2015. The operation took 90 min and the estimated blood loss was 100 mL. One day postoperative x-ray and CT scans showed a good position of the huge tear drop fracture fragment, the three cortical iliac bones, and anterior cervical plate (Figure 4). The patient was instructed to wear a cervical collar until their return to the department for a follow-up examination some 3 months after their surgery. The 3 months postoperative x-ray and CT scan showed a good position of the implant and bony fusion at the C2/3 segment (Figure 5).

## DISCUSSION

Generally, the mechanism of injury underpinning an axial tear drop fracture is regarded as hyperextension.^8^ HTDFA can lead to severe C2/3 intervertebral disc injury and obvious C2/3 instability. The surgical strategy should be aimed at restoring stability and cervical alignment but should also be effective, safe, and simple. How to treat HTDFA remains very controversial that many spinal surgeons remain unsure how to proceed. In this case, all of the spinal surgeons in the department took part in the discussion of treatment strategies and a literature search was also undertaken. We found that only a few cases of HTDFA were reported in previous studies (Table 1).^5–7,9–12^ Results from these case reports indicated that both conservative and surgical treatment methods seemed effective in the treatment of HTDFA. In addition, both anterior and posterior approaches were reported to be effective with regards achieving bone fusion.

**TABLE 1:**
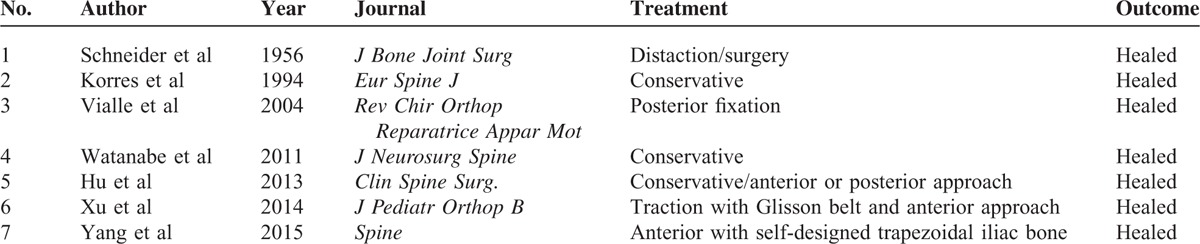
Summary of Huge Tear Drop Fracture of Axis Reported in Previous Studies

Hu et al retrospectively reviewed 16 patients with tear drop fracture of the axis and concluded that most patients with an extension tear drop fracture of the axis can be treated conservatively but large fragment size, displacement or angulation, intervertebral disc injury, neurologic deficit, or signs of instability are reasonable indications for surgical treatment.^11^ Opinions from the spinal surgeons in our department can also be grouped into three strategies: conservative treatment, anterior approach, or posterior approach. Some surgeons in our department preferred conservative treatment as they believed that the cervical spine was almost stable because its posterior structure remained undamaged; a halo-vest or skull traction was enough to prevent hyperextension; some surgeons in the department preferred the anterior approach because they believed that the large fragment (as in this case) was associated with C2/3 intervertebral disc injury and anterior stabilization was therefore a better method because it allowed direct reduction of the fracture fragment; some surgeons in the department suggested the posterior approach because they believed that posterior fixation may provide better stability.

After discussion, a surgical plan of anterior reduction, discectomy, and three cortical iliac bone grafts with instrumentation was reached. The reasons for this choice were as follows:

1. Even though the cervical spine was almost stable and conservative treatment was reported to be effective in some case reports, long-term external fixation such as that offered by use of a halo vest or skull traction would have caused discomfort for the patient. Conservative treatment may also be associated with chronic dysphagia as the large fracture fragment compresses the esophagus. C2/3 can only reach bony fusion with great difficulty and the C2/3 intervertebral disc was damaged in this patient so conservative treatment may have contributed to post-traumatic cervical kyphosis.
2. Although posterior fixation may provide better stability,^13^ patient positioning is more difficult, and it may result in iatrogenic damage to stabilizing posterior structures. Similarly, the posterior approach does not allow direct reduction of the fracture fragment and this may also cause chronic dysphagia and post-traumatic cervical kyphosis. The pedicles of the vertebral arch and posterior structure in this patient were not damaged and thus ensured cervical stability: a posterior approach may have caused iatrogenic damage to the posterior structure such as the ligaments and muscles and may have caused axial pain after surgery.
3. Anterior-only stabilization using anterior plating can be sufficient to stabilize single-level injuries, which is consistent with a recent biomechanical study.^14^ Hu et al believed that anterior stabilization is a better method because it allows direct reduction of the fracture fragment.^11^ Thus, the anterior approach can decrease the possibility of chronic dysphagia and post-traumatic cervical kyphosis.

This represented the application of a basic theory which is widely used in spinal surgery and associated research, namely: Denis three-column spine theory.^15,16^ A pure HTDFA is an injury to the anterior column, the cervical spine is stable under hyperflexion but not so stable when under hyperextension. Anterior reduction, discectomy, and three cortical iliac bone grafts with instrumentation can restore the stability of the cervical spine under hyperextension, maintain the disc height of C2/3, and realize bony fusion at C2/3. A noteworthy case presented by Yang et al was published recently.^7^ Yang et al performed surgery with a self-designed tricortical trapezoidal iliac bone, even they also used an anterior approach. Their case is interesting: through a classic anterior approach, they remove the huge fractured bone, burr the proximal end of the defect to form a trapezoidal shape, and then insert the self-designed tricortical trapezoidal iliac bone into the matching defect. They believe that this method can avoid the sliding of the grafted bone and restore normal intervertebral height and upper cervical alignment to a greater extent. Although we admit that their method is interesting and novel, we hold different opinions as outlined further:

1. In such a case of HTDFA, the aim of the anterior approach is to deal with the huge fracture fragment, and restore C2/3 disc height and upper cervical alignment. We did not think that the anterior part of the axis vertebra was required to bear the load of head and atlas; in such a case since the posterior part of the axis vertebra was undamaged and could bear the load if we inserted a cylindrical three cortical iliac bone graft into the C2/3 disc space. The cervical spine was stable under flexion and what was required was the reconstruction of its stability under hyperextension.
2. If we removed the fracture fragment, the periosteum would have been damaged which meant that the blood supply in this area was also damaged.
3. If we burred the proximal end of the defect to form a trapezoidal shape, the axis vertebra would be re-impaired, and this may have affected the strength of the axis vertebra and caused secondary fracturing, perhaps of the transverse axial type.
4. A self-designed tri-cortical trapezoidal iliac bone graft necessitated a much larger volume of iliac bone graft and a greater surgical trauma to the patient.
5. The fracture fragment was removed and thus wasted, the burring of the axis and iliac bone graft may waste more of the bone available. The whole complex procedure would have meant a longer surgical time demand and more blood loss in our opinion.

Another discussion point was the transnasal induction of general anesthesia during which the mouth of the patient would have been closed. This method of anesthesia can generate a larger mandibular angle which meant a better exposure space, which, in turn, would have rendered the procedure less complex.

## CONCLUSION

In summary, how to treat HTDFA remains controversial and the decision should be individualized while also considering: the fracture fragment size, displacement or angulation, intervertebral disc injury, neurologic deficit, or signs of instability and whether treatment will incorporate that for other fractures such as a fracture of the vertebral pedicle, a fracture of the vertebral lamina, or an odontoid fracture. Conservative treatment can be effective in most small, and pure, tear drop fractures of the axis but anterior reduction, discectomy, and bone grafting with instrumentation are warranted for most HTDFA. However, if HTDFA incorporates other complex fractures such as a fracture of the vertebral pedicle, a fracture of the vertebral lamina, or an odontoid fracture, anterior and posterior union surgery is recommended in these complex cases. After a 3-month postoperative follow-up examination, the outcome showed that anterior reduction, discectomy, and three cortical iliac bone grafts with instrumentation were effective, safe, and simple in the treatment of HTDFA. Of course, longer follow-up duration and more cases were warranted to verify this procedure.
